# Distinct CD4+ T cell signature in ANA-positive young adult patients

**DOI:** 10.3389/fimmu.2022.972127

**Published:** 2022-10-13

**Authors:** Flavia Dei Zotti, Chiara Moriconi, Annie Qiu, Anabel Miller, Krystalyn E. Hudson

**Affiliations:** Laboratory of Transfusion Biology, Department of Pathology and Cell Biology, Columbia University Irving Medical Center, New York, NY, United States

**Keywords:** ANA+, recent thymic emigrants (RTE), effector helper T cells (TH), mTOR, pre-autoimmune disease, ANA-, tolerance

## Abstract

Failure of immune tolerance can lead to autoantibody production resulting in autoimmune diseases, a broad spectrum of organ-specific or systemic disorders. Immune tolerance mechanisms regulate autoreactive T and B cells, yet some lymphocytes escape and promote autoantibody production. CD4+ T cell dysregulation, characterized by decreased or impaired regulatory cells (Tregs) and/or accumulation of memory and effector T cells such as TH17, plays a crucial role in the pathogenesis of these diseases. Antinuclear antibody (ANAs) testing is used as a first step for the diagnosis of autoimmune disorders, although most ANA-positive individuals do not have nor will develop an autoimmune disease. Studying the differences of T cell compartment among healthy blood donors, ANA-negative patients and ANA-positive patients, in which loss of tolerance have not led to autoimmunity, may improve our understanding on how tolerance mechanisms fail. Herein, we report that ANA-positive patients exhibit a distinct distribution of T cell subsets: significantly reduced frequencies of recent thymic emigrants (RTE) and naïve T cells, and significantly increased frequencies of central memory T cells, TH2 and TH17 cells; modulations within the T cell compartment are most profound within the 18-40 year age range. Moreover, CD4+ T cells in ANA-positive patients are metabolically active, as determined by a significant increase in mTORC1 and mTORC2 signals, compared to ANA-negative patients and healthy blood donors. No significant impairment of Treg numbers or pro-inflammatory cytokine production was observed. These results identify a unique T cell signature associated with autoantibody production in the absence of autoimmune disease.

## Introduction

Establishment and maintenance of immunological tolerance prevents development of autoimmune disease. Tolerance induction involves many mechanisms (e.g., deletion, anergy, etc.) and occurs in primary and secondary lymphoid organs. In most instances, autoreactive T and B cells are eliminated, become anergic, develop into regulatory T cells (Tregs) or are actively suppressed by Tregs (1, 2). When autoreactive lymphocytes escape tolerance mechanisms, it can lead to autoimmune pathology such as autoantibody production. Presence of abnormal levels of autoantibodies before disease onset can be clinically useful for the diagnosis of autoimmune disorders (3). Antinuclear antibodies (ANAs) are detected in up to 20% of the general population and are associated with autoimmune diseases, such as systemic lupus erythematosus (SLE), rheumatoid arthritis (RA), Sjögren’s syndrome (SjD) and multiple sclerosis (MS) (4–7). Although most ANA-positive individuals do not have an autoimmune disease and the probability of developing one during their lifetime is low, ANAs can appear many years prior to associated clinical features of disease (8–12). Because most cases of ANA positivity are not associated with pathology, studying immune cells from ANA-positive individuals may provide insight into how tolerance mechanisms to autoantigens fail.

T cell dysregulation is often associated with autoimmune diseases. Despite central and peripheral tolerance mechanisms, autoreactive T cells persist in the repertoire. Failure of T cell tolerance, and thereby initiation of autoimmune disease, can be due to environmental triggers, defective regulation, and/or genetic factors; these events have profound effects on the T cell compartment (13–15). For instance, decreased numbers or impaired function of Tregs can promote expansion of proinflammatory TH17 CD4+ T cells. Given their antagonistic functions, an imbalance of the Treg : TH17 CD4+ T cell ratio can signal active autoimmune disease; increased frequencies of TH17 cells is associated with autoimmune diseases due to their ability to secrete proinflammatory IL-17, promote B cell activation and elicit autoantibody production (16). Other T cell correlates with autoimmune disease include reduced frequencies of naïve T cells, accumulation of effector and memory T cells, mTOR activation, and elevated levels of inflammatory cytokines (17–21). Lastly, the aging process itself is associated with T cell dysregulation and increased risk of autoimmunity (22–25). Aging is accompanied by marked changes in the T cell compartment and chronic low levels of inflammation. Of note, age-related thymic involution leads to significant reductions in newly-generated naïve T cells, which stimulates compensatory peripheral expansion of T cells to maintain stable T cell levels (26, 27); this process may contribute to autoimmunity by inappropriately activating autoreactive T cell clones (28, 29). Additionally, thymic atrophy impairs efficient negative selection, leading to release of autoreactive T cells into the periphery (30). The cumulative effects of these changes within the T cell compartment have been characterized in autoimmune disease (17, 31); however, it is unclear how loss of humoral tolerance to autoantigens, in the absence of active autoimmune disease, affects the T cell compartment.

In many cases, production of ANAs is CD4+ T cell-dependent as autoreactive B cells have undergone antigen-driven clonal expansion and somatic mutation (32). Herein, we test the hypothesis that CD4+ T cells from ANA-positive patients are distinct from ANA-negative patients and healthy blood donors. To test this hypothesis, we analyzed and compared CD4+ T cells collected from ANA-positive patients, ANA-negative patients, and healthy blood donors. Because of age-related changes to the T cell compartment, data was analyzed based on age ranges. The most profound effects of ANA-positivity were observed in the 18-40 year age range whereby ANA-positive patients had significantly reduced frequencies of recent thymic emigrants (RTEs) and naïve T cells, compared to ANA-negative patients and healthy blood donors. In contrast, there was a significant increase in the frequencies of central memory, TH17, and TH2 CD4+ T cells detected in samples from ANA-positive patients, along with elevated plasma levels of IFNβ and IL-17, compared to controls. Unexpectedly, there were no differences observed in the frequency of Tregs or the ratio of Tregs to TH17 T cells. Finally, irrespective of age, CD4+ T cells from ANA-positive patients were significantly more metabolically active, compared to controls. Together, these data identify a T cell signature associated with production of ANAs that is distinct from age-related changes and unassociated with hospital admission.

## Materials and methods

### Human peripheral blood samples

Anonymized residual whole blood samples from individuals aged ≥ 18 years old were collected in EDTA and received from the Center for Advanced Laboratory Medicine at Columbia University or from consented healthy blood donors in an ongoing clinical trial (ClinicalTrials.gov #NCT02889133). Samples were de-identified and only age, sex, race, and ICD10 codes were collected (**Table 1** and **Supplementary Figure 1**). Human subject participation was approved by the Columbia University Institutional Review Board.

**Table 1:** Demographic characteristics of ANA-positive (ANA+), healthy blood donor (Healthy) and ANA-negative (ANA-) groups. Mean ± SEM was used.

	ANA+	Healthy	ANA-
Age (mean±SEM)	52±3.6	47±3	57±3.7
Sex (F/M)	9/11	15/17	10/10

### Sample processing and flow cytometry

Samples were spun at 400 x g for 10 minutes to separate plasma from white blood cells (WBCs) and red blood cells (RBCs). Plasma was collected for detection of cytokines (IL-1β, IL-6, IL12(p70), IL-8, IL-10, GM-CSF, TNFα, IFNγ, IFNα2, IFNβ, IFNλ1 and IFNλ2-3: LEGENDplex™ Biolegend #740390, and for IL-17A/F: Legend Max Biolegend #435807). Plasma samples were diluted 2-fold and analyzed per manufacturer’s instructions. Peripheral blood mononuclear cells (PBMCs) were isolated from whole blood by Ficoll density gradient centrifugation (Ficoll-Paque™ PLUS GE Healthcare #17-1440-02). Samples were washed with FACS buffer (phosphate buffered saline (PBS) supplemented with 2% fetal bovine serum and 0.4% of Ethylenediaminetetraacetic acid (EDTA)), stained for surface and intracellular markers for 30 minutes at 4°C with antibodies specific for CD4 (RPA-t4), CD3 (SK7), CD45RA (HI100), CD45RO (UCHL1), CD25 (CD25-4E3, BC96, M-A251), CCR7 (G043H7), CD31 (WM-59), FoxP3 (PCH101), CD194 (CXCR4;1G1), CD183 (CXCR3;1C6), CD196 (CCR6;11A9), CCR10 (1B5), CD185 (CXCR5;MU5UBEE), CD279 (PD1;J105), CD278 (ICOS;ISA3), p-Akt (SDRNR) and p-S6 (D57.2.2E). Staining for intracellular FoxP3, p-Akt and p-S6 was performed according to the protocol from Invitrogen using eBioscience™ FOXP3/Transcription kit (#00552300). Samples were collected using an Attune NxT flow cytometer (ThermoFisher) and data were analyzed with FlowJo software (BD Biosciences).

### Statistical analysis

Subjects were divided into age groups (18-40, 40-60 and 60-80 years old) *a priori* due to the acceleration of thymic atrophy and cessation of active thymopoiesis after 40 years of age (33, 34) and the increase in immunosenescence that can alter T cell functionality after 60 years of age (35, 36). Between-group differences were compared using an analysis of variance (ANOVA) with Sidak’s multiple comparison test and age as a categorical variable. Analyses were performed using Prism, version 9 (GraphPad Software, Inc.). For added rigor, the frequency of each T cell subset was also analyzed by multivariable linear regression incorporating age as a continuous variable and patient type (i.e., ANA-positive, ANA-negative and Healthy blood donors) as a categorical variable using SAS studio version 3.8 (SAS Institute Inc.). For comparisons that yielded different statistical results between ANOVA and multivariable linear regression analyses, additional **supplemental figures** were included. A p-value less than 0.05 was considered significant.

## Results

### Reduced frequencies of detectable recent thymic emigrants (RTEs) in young ANA-positive patients

**Figure 1:**
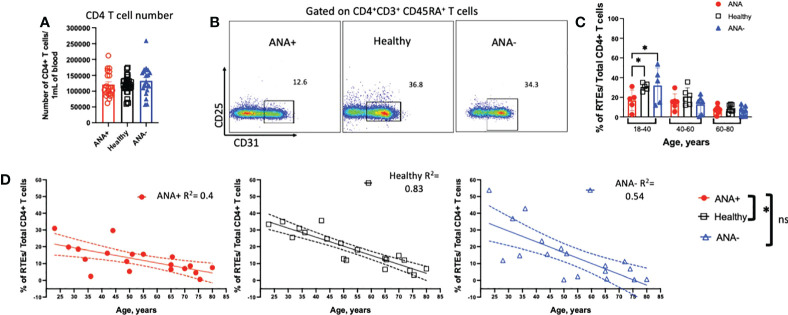
Young ANA-positive patients have fewer RTEs. PBMCs were isolated from whole blood and stained with antibodies to identify recenty thymic emigrants (RTEs). **(A)** The absolute number of CD4+ T cells per 1mL of blood was determined. **(B)** Representative flow plots of CD4+CD3+CD45RA+CD45RO-CD31+CD25- RTEs in ANA-positive patients, healthy blood donors, and ANA-negative patients. Subjects were analyzed in groups based on age ranges: 18-40 (young), 40-60 (middle), and 60-80 (old) years old. **(C)** The frequency of RTEs of total CD4+ T cells was calculated and data are shown as mean ± SEM and analyzed by a one-way ANOVA test followed by Tukey’s multiple comparison test; *p<0.05. **(D)** The correlation between the frequency of RTEs and age was plotted. Linear regression analysis was used for all three groups and R2 are reported. Each data point reflects one subject (ANA-positive = 20, ANA-negative = 20, healthy blood donors = 32). Subjects were analysed using multiple regression analysis incorporating age as a continuous variable and type of blood donors (ANA-positive, ANA-negative, healthy blood donors) as a categorical variable followed by Tukey’s multiple comparison test; *p<0.05; ns, non significant.

Blood samples were collected from patients previously screened for antinuclear autoantibodies (ANAs) (N=40; 20 each of ANA-positive and ANA-negative) and healthy blood donors (N=32; **Table 1** and **Supplemental Tables S1, 2**). T cell tolerance prevents many autoimmune diseases; thus, we hypothesized that T cell dysregulation would be evident in patients with prior ANA-positive results. Peripheral blood mononuclear cells (PBMCs), isolated from whole blood, were stained to identify CD4+ T cells. Absolute numbers of CD4+ T cells were unchanged in ANA-positive, compared to ANA-negative and healthy blood donors (**Figure 1A**). RTEs are the youngest peripheral T cells susceptible to peripheral T cell tolerance mechanisms. Due to thymic involution, the number of RTEs decrease with age, with a notable reduction in thymic output after 40 years of age (34, 37); as such, samples were evaluated in groups based on age ranges: (i) 18 to 40 years (young), (ii) 40 to 60 years (middle), and (iii) 60 to 80 years (old). RTEs, defined phenotypically as CD4+CD3+CD45RA+CD45RO-CD31+CD25-, were readily identified by flow cytometry (**Figure 1B**, gating strategy **Supplemental Figure 1**). Young ANA-positive patients had significantly fewer RTEs, compared ANA-negative patients and healthy controls (**Figure 1C**, p<0.05). To increase the statistical rigor, ANA-positive, ANA-negative and healthy controls were analysed with a multiple regression analysis with age as a continuous variable. ANA-positive had a significant reduction in the frequency of RTEs compared to healthy controls (statistical analysis **Figure 1D**). No significant differences in RTEs were noted with either statistical analysis approach between young ANA-negative patients and healthy controls. Consistent with prior observations (38, 39), the frequency of RTEs declined with age in ANA-positive patients, ANA-negative patients and healthy blood donors (**Figure 1D**; respectively R^2 =^ 0.44, R^2 =^ 0.54 and R^2 =^ 0.83). These data demonstrate that the RTE population in patients with ANAs is reduced compared to healthy controls, suggesting these patients may have perturbed differentiation into naïve, effector, and memory CD4+ T cell subsets.

### Altered peripheral T cell populations detected in ANA-positive patients

**Figure 2:**
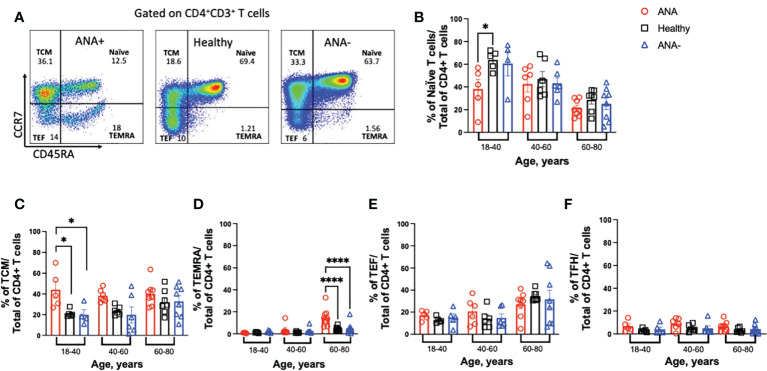
*Young ANA-positive patients have reduced naïve and increased differentiated CD4+ T cells.* PBMCs were isolated from whole blood and stained with antibodies to identify T cell subsets. **(A)** Gating strategy to visualize naïve, T central memory (TCM), terminally differentiated effector memory (TEMRA), and T effector (TEF) CD4+ T cells by flow cytometry. The frequencies of **(B)** naïve, **(C)** TCM, **(D)** TEMRA, **(E)** TEF, and **(F)** T follicular helper (TFH) T cell subsets of total CD4+ T cells was determined in ANA-positive patients, ANA-negative patients, and healthy blood donors. Each data point reflects one subject (ANA-positive = 20, ANA-negative = 20, healthy blood donors = 32). Data are shown as mean ± SEM and analyzed by a one-way ANOVA test followed by Sidak’s multiple comparison test; *p<0.05, ****p<0.0001.

In response to antigen exposure with co-stimulation, naïve T cells proliferate and differentiate into effector and memory T cells (40). To determine how generation of ANAs effected T cell subsets, CCR7 and CD45RA were used to discriminate naïve (CD45RA+CCR7+), central memory (TCM; CD45RA-CCR7+), effector (TEF; CD45RA-CCR7-) and terminally differentiated effector memory (TEMRA; CD45RA+CCR7-) CD4+ T cell subsets (**Figure 2A**) (41). Young ANA-positive patients had significant reductions in the frequency of circulating naïve T cells and increased percentages of TCM, compared to ANA-negative patients and healthy blood donors (**Figures 2B, C**, p<0.05). Based on prior reports (42), we expected the frequency of TEMRA cells T cells to increase with age. In ANA-positive and healthy blood donors, TEMRA T cells showed moderate correlation with age (**Figure 2D** and **Supplemental Figure 2**; R^2 =^ 0.44 and R^2 =^ 0.38, respectively). No correlation between TEMRA T cells and age was observed in samples from ANA-negative patients (R^2 =^ 0.09). Comparing the frequency of TEMRA T cells in older individuals, the percentages were significantly increased in ANA-positive patients, compared to ANA-negative patients and healthy blood donors (**Figure 2D**). Analysis of other differentiated T cell subsets revealed no differences in the percentages of TEF or T follicular helper (TFH) CD4+ T cells (**Figures 2E, F**, gating strategy shown in **Supplemental Figure 3**).

### Qualitative differences in TEF and TFH CD4+ T cells from ANA-positive patients

**Figure 3:**
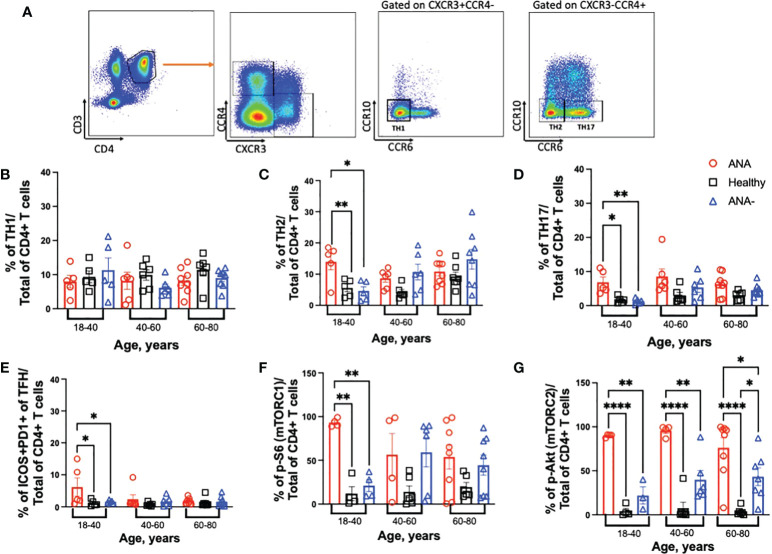
*Young ANA-positive patients have increased frequencies of TH2 and TH17 CD4+ T cells that are metabolically active.* PBMCs were isolated from whole blood collected from ANA-positive patients, ANA-negative patients, and healthy blood donors. Cells were stained with antibodies to identify CD4+ T helper (TH) subsets, activated TFH, and mTOR activation. **(A)** Representative gating strategy to visualize TH1, TH2 and TH17 CD4+ T cells. The frequencies of **(B)** TH1, **(C)** TH2, **(D)** TH17 and **(E)** ICOS+PD1+ TFH CD4+ T cells of total CD4+ T cells was determined. mTOR activation was evaluated by **(F)** p-S6 (marker for mTORC1) and **(G)** p-Akt (marker for mTORC2) on total CD4+ T cells. Each data point reflects one subject (ANA-positive = 20, ANA-negative = 20, healthy blood donors = 32). Data are shown as mean ± SEM and analyzed by one-way ANOVA test followed by Sidak’s multiple comparison test; *p<0.05, **p<0.01, ****p<0.0001.

Upon activation, CD4+ T cells differentiate into specialized effector T helper (TH) subsets, characterized by distinct cytokine profiles and function. To test for differences in the distribution of TH subsets, TH1 (CCR4-CXCR3+CCR10-CCR6-), TH2 (CCR4+CXCR3-CCR10-CCR6-), and TH17 (CCR4+CXCR3-CCR10-CCR6+) CD4+ T cells were identified in PBMCs (gating strategy shown in **Figure 3A**). In all age groups, similar frequencies of TH1 CD4+ T cells were observed between ANA-positive patients, ANA-negative patients, and healthy blood donors (**Figure 3B**). In contrast, increased frequencies of TH2 and TH17 CD4+ T cells were observed in young ANA-positive patients, compared to ANA-negative patients and healthy blood donors (**Figures 3C, D**). Differences in TH subset distribution was not correlated with a particular disease state at the time of whole blood collection (**Supplemental Figure 4**). Finally, a specialized subset of circulating TFH CD4+ T cells, co-expressing PD-1 and ICOS, has been shown to reflect activated memory TFH and can facilitate B cell differentiation into plasma and memory cells (43). Young ANA-positive patients also had significantly higher percentages of PD1+ICOS+ TFH cells, compared to ANA-negative patients and healthy controls (**Figure 3E**, gating strategy shown in **Supplemental Figure 3**).

The mTOR pathway plays a key role in shaping T cell differentiation by differential activation of mTORC1 and mTORC2. And, mTOR activation in T cells has been noted during the development of several autoimmune diseases such as SLE, MS and RA (19, 44–47). To test whether CD4+ T cells were metabolically active, mTORC1 and mTORC2 activation was determined by detection of phosphorylated (p) -S6 and -Akt, respectively. Young ANA-positive patients had significantly higher frequencies of p-S6, compared to ANA-negative patients and healthy blood donors (**Figure 3F**); no differences were noted in older cohorts. Frequencies of TH2 cells and mTORC1 in ANA-positive patients were significantly increased only compared healthy controls, after multiple linear regression analysis with age as a continuous variable were performed. In contrast to ANA-positive patients, TH2 cells and mTORC1 accumulate with age in ANA-negative patients (**Supplemental Figures 5A, B**). CD4+ T cells from ANA-positive patients in all age groups had significantly elevated frequencies of p-Akt, compared to ANA-negative and healthy blood donors (**Figure 3G**). Together, these data demonstrate that CD4+ T cells from ANA-positive patients are metabolically activated and have preferentially differentiated into TH17 effector T cells.

### ANA production does not correlate with reduction in regulatory T cells

**Figure 4:**
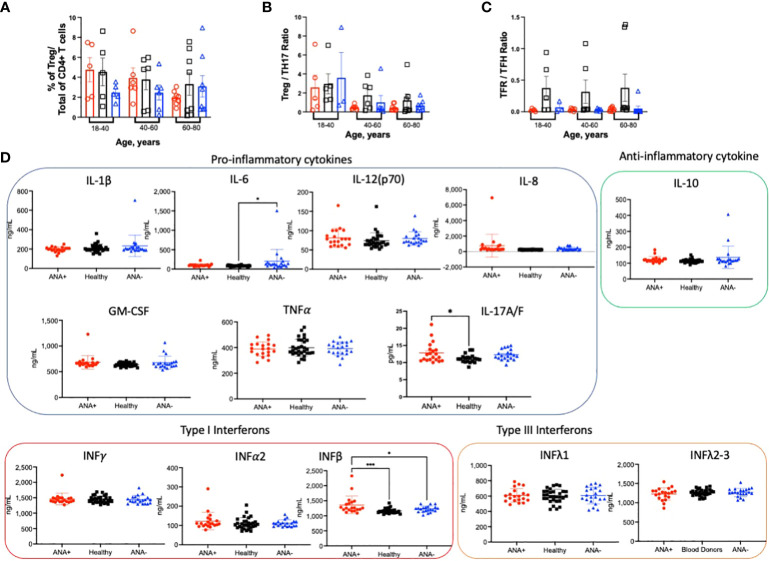
*ANA-positivity does not correlate with changes in regulatory CD4+ T cells*. PBMCs were isolated from whole blood collected from ANA-positive patients, ANA-negative patients, and healthy blood donors. Cells were stained with antibodies to identify **(A)** FoxP3+CD25+ regulatory T cells (Tregs). The ratios between **(B)** Tregs and TH17 and **(C)** T follicular regulatory (TFR) and TFH CD4+ T cells were calculated. **(D)** Plasma cytokines were measured by multiplex immunoassay. Each data point reflects one subject (ANA-positive = 20, ANA-negative = 20, healthy blood donors = 32). Data are shown as mean ± SEM and analyzed by a one-way ANOVA followed by Sidak’s multiple comparison test; *p<0.05, ***p<0.001.

An imbalance between regulatory and inflammatory immune cells and a pro-inflammatory environment are associated with autoimmune diseases (10, 17, 18, 21). To assess whether regulatory T cell (Treg) frequencies were altered in PBMCs from ANA-positive patients, Tregs were identified by staining with antibodies against CD25 and FoxP3. The percentage of Tregs was similar in every subject group and in all age ranges (**Figure 4A**). Because we observed an increased frequency of proinflammatory TH17 CD4+ T cells, we calculated the Treg: TH17 CD4+ T cell ratio. Indeed, a decreased ratio correlates with many autoimmune diseases (48, 49). No significant difference in the Treg : TH17 ratio was observed; however, there was a trend of decreasing ratios observed upon aging (**Figure 4B**). A distinctive subset of FoxP3+ Treg cells, defined by CXCR5+ expression, are bona fide circulating T follicular regulatory cells (TFR). Presence of TFR in blood is indicative of ongoing humoral activity and significantly increased in patients with Sjögren syndrome (50, 51). There was no significant difference in the ratio of TFR within TFH T cells for any age group (**Figure 4C**). However, multiple linear regression analysis showed a significant decreased in the ratio of TFR : TFH T cells in both type of patients (ANA-positive and ANA-negative) compared healthy blood donors (**Supplemental Figure 5C**). The environment was studied by quantifying circulating cytokines with protective or deleterious role. Of the cytokines analyzed, only IFNβ was significantly higher in ANA-positive patients, compared ANA-negative patients and healthy blood donors (**Figure 4D**). Elevated levels of IL-17 were detected in ANA-positive patients, compared to healthy blood donors. Of note, higher data points observed in ANA-positive and ANA-negative patients correspond to different individuals. Together, these data suggest that ANA-positive patients do not have decreased Treg subsets nor can they be defined by a particular inflammatory cytokine profile.

## Discussion

The data presented herein demonstrate that the T cell compartment in ANA-positive patients, in which tolerance mechanisms have failed but have not led to active autoimmune disease, are distinct from ANA-negative patients and healthy blood donors. The most significant differences were observed in young 18-40 year old individuals whereby ANA-positive patients had significantly reduced numbers of naïve T cells and increased frequencies of effector T helper cell populations such as TH17 and TH2 CD4+ T cells. Unexpectedly no significant differences were observed in Tregs or the ratio of Treg : TH17 CD4+ T cells between groups. More generalizable to all age groups analyzed, ANA-positive patients had metabolically active CD4+ T cells and elevated levels of plasma IFNβ and IL-17 cytokines, compared to ANA-negative patients and healthy blood donors. Together, these data show that generation of ANAs, without active autoimmune disease, correlates with distinct changes in the CD4+ T cell compartment in young patients.

While age is not a significant predictor of autoantibody positivity (52), some studies report that the prevalence of ANAs increases with age in absence of autoimmune disease (4, 53). However, the aging process does siginificantly impact the T cell compartment due to antigen exposure, low-grade inflammation, cellular senescence and thymus involution (54). By analyzing subjects based on age ranges, significant changes within T cell subsets were observed. In particular, the frequency of RTEs progressively decreased with age in all groups, as expected (37). While age was a good predictor of the frequency of RTEs in healthy blood donors (i.e., R^2 =^ 0.83), it was not a strong correlate for ANA-positive or ANA-negative patients. This could be due to other, underlying medical conditions as these patients were currently in the hospital. Of interest, however, the presence of ANAs in young patients significantly reduced the frequency of CD4+ RTEs. RTEs are the youngest peripheral naïve T cells and are susceptible to tolerance induction (55); studies have shown that tolerance failure can induce autoimmune disease. Thus, the observed decreased frequencies of RTEs in ANA-positive patients may be due to premature differentiation into effector T cells and reflect tolerance failure. In general, aging also led to decreased frequencies of naïve T cells and an accumulation of TCM and TEF CD4+ T cells, as expected. However, consistent with the observation that young ANA-positive patients had decreased naïve T cell populations, these patients had a significant increase in TCM T cells; thus, there was no observed increase in TCM upon aging in this group. Finally, terminally differentiated effector memory (TEMRA) T cells that re-express CD45RA after antigen-stimulation have been documented to accumulate with age. TEMRA T cells exhibit multiple characteristics of senescent cells including DNA damage, low proliferative potential, high levels of reactive oxygen species (ROS) and can also be cytotoxic as it has the ability to secrete inflammatory cytokines such as IFNγ and TNFα (56, 57). In our study, the frequency of TEMRA cells slightly increase with age in all groups. However, older ANA-positive individuals have significantly increased percentages of TEMRA T cells, compared to ANA-negative patients and healthy controls; these data suggest that an accumulation of TEMRA T cells may be due to, or the result of, autoimmunity. And, although there is significant accumulation of differentiated T cell subsets in young ANA-positive patients, the data show that these T cells do not become senescent. Further studies will be required to investigate the role of ANAs in TEMRA T cell differentiation. Together, these data show that the presence of ANAs is associated with alterations in the T cell compartment, especially in 18-40 year old patients.

T cell lineage commitment can be influenced by the metabolic status of T cells. Antigen recognition triggers robust mTOR activation, which drives the differentiation of naive CD4+ T cells into the TH1, TH2 and TH17 cell effector lineages, while also inhibiting the induction of Tregs and T cell anergy (19, 58, 59). Our data showed that both mTOR complexes, mTORC1 and mTORC2, were significantly increased in CD4+ T cells of ANA-positive patients. While mTORC1 activation can promote expansion of TH1 and TH17 subsets, mTORC2 activation leads to TH2 differentiation; as increased frequencies of mTORC1 and mTORC2 activation was observed in CD4+ T cells from young ANA-positive patients, this could explain the elevated numbers of TH17 and TH2 T cell subsets. However, despite the fact that ANA-positive subjects had higher frequencies of TH2 cells and mTORC1 activation, compared healthy blood donors in all age groups, in ANA-negative patients these frequencies increased with age and are comparable to ANA-positive subjects suggesting that diseases state alone can influence T cell differentiation and activation. Increased frequencies of TH2 and TH17 CD4+ T cells are associated with autoimmune diseases such as SLE, RA and systemic sclerosis (60–62). The frequency of T helper cells can be altered by the presence of other pathologies such as inflammation or immunodeficiency (63, 64). However, no significant correlation between diseases and distribution of T helper cells were observed. Despite the high frequencies of CD4+ T cells with mTOR activation, it was surprising that no overt alterations in Tregs were noted. However, Tregs were only determined by phenotype and were not assayed for functional capabilities. mTORC1 and mTORC2 can also promote differentiation of TFH cells (65–67), another specialized CD4+ T subset involved in the pathogenesis of autoimmune diseases (68). Both TFH and CD4+ helper T cells interact with and provide help to B cells to induce differentiation and antibody production (69). It has been shown that increased frequencies of circulating TFH are detectable in patients with autoimmune diseases (70). PD1 and ICOS are co-stimulatory molecules essential for the development and function of TFH. In our study, while the frequency of TFH cells was similar in all subjects, the frequency of circulating PD1+ICOS+ TFH cells in ANA-positive patients was higher than that in ANA-negative and healthy controls. Increased numbers of ICOS+ TFH cells in peripheral blood are detectable in patients with autoimmune diseases including SLE, SjD, RA, and autoimmune thyroid diseases (71, 72). ICOS plays an important pro-inflammatory role in the late effector phase and T memory-dependent B cell response (73) and PD1 is expressed on activated T cells (74). Taken together, we speculate that high levels of mTOR activation in T cells from ANA-positive patients contribute to autoantibody production by expanding effector T cells and PD1+ICOS+ TFH, which may eventually promote future development of autoimmune disease.

Dysregulated immunoregulation also contributes to failure of immune tolerance. Prior studies have shown that development of autoimmune diseases is influenced by decreased numbers or function of Tregs and a corresponding expansion of effector cells, reflecting an imbalance between immunoregulatory and inflammatory cells. The decreased ratios of Treg : TH17 and TFR : TFH have been proposed as useful metrics for active autoimmune diseases such as SjD and RA (50, 75, 76). However, there is disagreement between studies on whether these ratios differ between ANA-positive patients, ANA-negative patients, and patients with an autoimmune disease (77, 78). Our data showed no significant differences in the ratios of Treg : TH17 or TFR : TFH between ANA-positive and ANA-negative patients; both patient groups had significantly decreased ratio of TFR : TFH compared to healthy blood donors. Thus, the underlying factor(s) that promote naïve T cell differentiation into TCM or TEF remain unclear. Although no numerical differences were observed in Tregs from ANA-positive patients, there was a significant increase in proinflammatory TH17 T cells, an observation consistent with other publications (79, 80). TH17 T cells secrete proinflammatory cytokine IL-17, which is detectable in plasma of patients with autoimmune disease. Increased inflammatory cytokines are also reported in ANA-positive patients (77), albeit to a lesser degree than those found in patients with autoimmune disease (17, 77, 81). Of the cytokines measured in our study, only IFNβ and IL-17 were elevated in plasma from ANA-positive patients, compared to ANA-negative patients and healthy blood donor controls. The absence of an overt pro-inflammatory cytokine signature in plasma from ANA-positive patients is consistent with the lack of active autoimmune disease. Additionally, IFNβ was described with a protective role in acute viral infections and deleterious role in bacterial infection (82); in autoimmune diseases, IFNβ is used as an effective treatment to reduce recurrence in multiple sclerosis (83) by activating regulatory T cells thereby limiting the generation of TH17 response and modulating pro-inflammatory mediators (84). Moreover, IFNβ is involved in down-regulating the inflammatory response by inhibiting antigen presenting cells, increasing Treg activity, reducing the ability of B cells to present antigens (85) and shifting Th1/Th2/Th17 polarization to an anti-inflammatory state (86, 87), this could be a protective mechanism to prevent disease onset. Together, these data highlight that, although differences in the T cell compartment were evident in ANA-positive patients, there was no significant alteration observed in regulatory T cell subsets nor strong upregulation of multiple pro-inflammatory cytokines, as observed in active autoimmune disease.

The current study has several limitations. Each patient (both ANA -positive and -negative) was in the hospital for an organ transplant or had been diagnosed with other diseases (e.g., heart failure, cancers, hypertension, kidney failure, fever, and respiratory issues) which can, in and of itself, elicit different immune responses that impact T cells. Additionally, the samples analyzed in this study were de-identified and data regarding medical treatment, which would influence immune cells, were not collected. Moreover, although demographic data were collected, subjects were not matched by age, sex, or ethnicity. Finally, no patient was diagnosed with an autoimmune disease at the time of blood collection and, due to the nature of the study design, it is impossible to perform a long-term follow up to assess longlasting effects T cell compartment changes and determine the rate of autoimmune disease.

Herein, we show that CD4+ T cells from ANA-positive patients are distinct from ANA-negative patients and healthy blood donors. Although ANA-positive patients do not have an active autoimmune disease, the production of ANAs correlated with marked changes in the T cell compartment. As such, these data provide new insights on the distribution of T cell subsets, mTOR activation, and cytokine production in the absence of pathogenic autoimmune responses. Investigating these mechanisms in a longitudinal study of ANA-positive subjects would improve our knowledge in the development of T cell distribution at the onset and upon progression of autoimmune disease.

## Data availability statement

The raw data supporting the conclusions of this article will be made available by the authors, without undue reservation.

## Ethics statement

This study was reviewed and approved by the Columbia University Institutional Review Board. Written informed consent was obtained from all participants for their participation in this study.

## Author contributions

FD and KH designed the studies and experiments. FD, CM, AQ, and AM collected and processed samples from subjects and volunteers. FD stained samples, collected data, and performed data analysis. FD and KH wrote the manuscript. All authors participated in data interpretation, revised the manuscript, and approved of the submitted version. All authors contributed to the article and approved the submitted version.

## Funding

These studies were supported, in part, by National Institutes of Health National Heart, Lung and Blood Institute R01HL133325 (KH).

## Acknowledgments

The authors would like to thank Sylvia Parker Jones and Lin Wang for the invaluable support in providing samples from human patients and volunteers. We also thank Eldad A. Hod for statistical support for these studies.

## Conflict of interest

Although unrelated to the contents of this manuscript, author KH has a sponsored research agreement with Alpine Immune Sciences.

The remaining authors declare that the research was conducted in the absence of any commercial or financial relationships that could be construed as a potential conflict of interest.

## Publisher’s note

All claims expressed in this article are solely those of the authors and do not necessarily represent those of their affiliated organizations, or those of the publisher, the editors and the reviewers. Any product that may be evaluated in this article, or claim that may be made by its manufacturer, is not guaranteed or endorsed by the publisher.
