# Supplementary material for: Distinct CD4+ T cell signature in ANA-positive young adult patients
Source: Front Immunol. 2022 Oct 13;13:972127. doi: 10.3389/fimmu.2022.972127 (PMC9608560; doi:10.3389/fimmu.2022.972127)
Supplement: Supplementary file 1 [file DataSheet_1.docx]

**Supplemental Figure Legends**

Supplemental Table 1. Demographic and clinical characteristics of each ANA-positive patient.

Supplemental Table 2. Demographic and clinical characteristics of each ANA-negative patient.

Supplemental Figure1. Representative flow plots of the gating strategy to visualize RTE T cells, defined as CD4+CD3+CD45RA+CD45RO-CD31+CD25-.

Supplemental Figure2. Correlation between the frequency of TEMRA CD4+ T cells detectable in blood and subject age. Linear regression analysis was used for all three groups and R^2^ are reported.

Supplemental Figure3. Representative flow plots of the gating strategy to visualize TFH T cells, defined as CD3+CD4+, CD45RA-, CXCR5+ and activated ICOS+ PD1+ TFH cells.

Supplemental Figure 4. Frequencies of TH1, TH2 and TH17 in ANA-positive and ANA-negative patients. Each subject was categorized based on the diagnosed disease: green for individuals presenting fever and/or inflammation that could suggest activation of the immune response, blue for patients with immunodeficiency, cancer, bone marrow or organ transplants that could associate with immunosuppression and black for patients with other diseases that can influence the immune response (e.g. heart failure, chronic respiratory failure, cardiomyopathy, renal dialysis, obesity, symptoms and signs involving the circulatory and respiratory systems).

Supplemental Figure 5. Multiple linear regression analysis of A) TH2 T cells, B) p-S6 (mTORC1) and C) of the ratio between TFR and TFH CD4+ T cells in ANA-positive, healthy and ANA-negative blood donors with age as a continuous variable and type of blood donors (ANA-positive, ANA-negative, healthy blood donors) as a categorical variable followed by Tukey’s multiple comparison test; *p<0.05.
